# Hypoglycemia and safe driving

**DOI:** 10.4103/0256-4947.72268

**Published:** 2010

**Authors:** Almoutaz A. Ahmed

**Affiliations:** From the Gurayat Diabetes Center, Gurayat North, Saudi Arabia

## Abstract

The lack of awareness of the effects of hypoglycemia on safe driving is a real issue for diabetic patients and a challenge for health care providers. Taking the form of questions and answers, this review addresses the issue of road traffic accidents and drivers with type 1 diabetes mellitus. While there is little evidence showing higher accident rates among diabetic drivers, there is research indicating that hypoglycemia compromises driving performance, resulting in slower response times and reduced cognitive function. Unawareness of an early fall in plasma glucose is another important issue that affects some diabetic drivers. The driver with type 1 diabetes is obliged to check their blood glucose before driving. The physician’s duty is to familiarize the patient with the risk of hypoglycemia. If hypoglycemic unawareness is present, the physician should advise the patient to stop driving until the condition is reversed. The doctor should consider informing authorities if he concludes there is a risk and the driver cannot be persuaded to stop driving.

In this review, the subject of hypoglycemia and safe driving is presented in the form of a series of questions. These are questions that might be asked of a diabetologist called as an expert witness in a trial involving a driver with type 1 diabetes who has caused an accident by dangerous driving.

## Q 1. What is the effect of hypoglycemia on mental status?

Investigations into the effect of glucose on performance have been conducted in humans. Some studies have explored the effect of glucose on learning, memory and mood in school children; on attention, memory and decision making in college students; and on memory in adults. Benton, Parker and Donohoe^1^ provided a useful overall review of the effect of blood glucose on cognitive functioning. They discussed the widely accepted view that hypoglycemia causes physical and psychological symptoms associated with disruption of cognitive function.^1^ Hypoglycemia has been found to induce adrenergic symptoms such as nervousness and tremor, as well as tiredness, confusion and retarded mental function.^2^ The first signs and symptoms of low blood sugar can begin to occur when serum glucose drops under 70 mg/dL, although this varies from person to person. Comi stated that complete cognitive recovery may lag for 30 to 45 minutes behind restoration of normal blood glucose levels.^3^ Safe driving requires constant integration of mental function (visual and auditory processing, or motor skills; reasoning, logic or problem solving).

Certain visual functions are affected at low blood glucose levels, e.g., detection of visual change and movement and the reaction time to visual stimuli.^4^ Performance of visual tasks requiring more involved processing was found to be adversely affected at low blood glucose levels. Also, decision making ability based on auditory processes was likewise impaired at low blood glucose levels.^5^ Aspects of attention are affected at low blood glucose levels: speed of performance on attention-requiring tasks was reduced when blood glucose levels were low, and subjects then became slower in their reaction response.^6^

Reaction time in the performance of vigilance tasks, detection of auditory or visual tones and tracking tasks was significantly lower at low blood glucose levels.^7^ Planning performance was slower, and mental flexibility and tracking were impaired at low blood glucose levels.^8^ Low blood glucose levels significantly impaired complex task performance, assessed by driving simulation.^9^ Steering control, speed control and braking were negatively affected at low blood glucose levels. Accuracy of performance was preserved at the expense of speed in these tasks, with very slow driving being demonstrated. Interestingly, patients with hypoglycemia are not always aware of their impairments.^10^

## Q2. For a driver with type 1 diabetes, are there risks associated with driving?

No randomized controlled studies (which provide the strongest evidence) exist. Sophisticated driving simulator studies with demanding scenarios showed that driving in itself is a significant stressor that is associated with greater autonomic symptoms, higher epinephrine levels and higher glucose need.^11^ Cox, Gonder-Frederick, Kovatchev, and Clarke found that actual driving was associated with a higher dextrose infusion rate (*P*=.02), more autonomic symptoms (*P*<.05), increased heart rate (*P*<.001), a trend toward greater epinephrine release (*P*=.09) and more frequent hypoglycemic self-treated (*P*<.001).^11^

Driving puts individuals with type 1 diabetes at increased risk of hypoglycemia.^11^ Simulator performance studies showed that during hypoglycemia (even if mild with blood glucose between 4.0 and 3.4 mmol/L), persons with type 1 diabetes engage in some form of less safe driving (driving across the midline, speeding and/or inappropriate braking).^2^ The exact blood glucose range at which driving is impaired and the exact driving parameters disrupted were found to be idiosyncratic.^10^

Depression of CNS activity (evidenced by increased theta wave activity in EEG) occurs at moderate hypoglycemia (3.3-2.8 mmol/L).^10^ Moreover, such studies revealed that 23% to 43% of type 1 diabetes patients with awareness of impaired hypoglycemia (based on epinephrine level and symptoms score assessed by various neuropsychological tests) failed to decide not to drive during hypoglycemia, increasing the chance of dangerous driving.^12^,^13^

Functions that are mainly affected by hypoglycemia include rapid decision making, sustained attention, analysis of complex visual stimuli and hand-eye coordination.^14^ In patients with type 1 diabetes and repeated hypoglycemia, there is marked deterioration of performance in tests of short-term, delayed and working memory for both verbal and nonverbal material.^15^

## Q3. Is there an increased risk of accidents among diabetic drivers?

This has been controversial, but a recent retrospective study showed that significantly more drivers with type 1 diabetes made at least one auto crash over the preceding 2-year period as compared to drivers with type 2 diabetes or nondiabetic spouses (19% vs. 12% vs. 8%, respectively; *P*<.001).^7^ Also, significantly more of those drivers with type 1 diabetes reported episodes of mild symptomatic hypoglycemia, hypoglycemic stupor and required assistance while driving due to development of hypoglycemia than the type 2 diabetic or nondiabetic drivers.^16^ Type 1 diabetes drivers also reported twice as many crashes as their nondiabetic spouses.^16^ Type 1 diabetes drivers who did not self-treat had almost three times the crash rate. The three main factors that significantly contributed to an increased probability of crashes were experiencing more episodes of hypoglycemic stupor while driving, less frequent self-monitored blood glucose before driving and taking insulin by subcutaneous injections rather than an insulin pump.^16^

## Q4. Can you ascertain that a type 1 diabetic was in an accident suffering from a hypoglycemic attack?

It is difficult to ascertain, but clues to significantly increased possibility of a hypoglycemic stupor while driving in type 1 diabetes are accepting a lower blood glucose as a threshold for deciding not to drive and suffering more frequent episodes of mild symptomatic hypoglycemia while driving.^16^ However, a diabetic with tight glycemic control (evidenced by glycated hemoglobin) is at more risk of hypoglycemia. The presence of hypoglycemia unawareness indicates the occurrence of previous severe hypoglycemia and raises the possibility that our patient suffered an attack while driving. The blood glucose in relation to driving and the time factor for development of events (in view of the recommendations) is necessary to judge further.

## Q5. What must have happened before getting into a state of hypoglycemic unawareness?

Hypoglycemia unawareness is when a subject is unaware of an early fall in plasma glucose concentration and does not recognize the warning neuroglycopenic symptoms and fails to compensate by eating to prevent progression to severe hypoglycemia. The syndrome is the result of deficient sympathetic neural and adreno-medullary responses to falling glucose levels. Hypoglycemia unawareness can occur in a longstanding diabetic patient who has a defect in secretion of counter-regulatory hormones. It develops if there are frequent bouts of hypoglycemia (level below 3.0 mmol/L).^17^

## Q6. Can a person know that he himself is prone to hypoglycemic unawareness?

By definition, a patient with hypoglycemia unawareness cannot recognize this state; full assessment can be completed by interviewing persons close to the patient in everyday life. Health education, self-monitoring of blood glucose can help, especially after exercise and during sleep.^17^

## Q7. Can people go straight into hypoglycemic unawareness without previously experiencing a symptomatic episode of hypoglycemia? How many such previous symptomatic episodes may occur before the development of hypoglycemia unawareness?

Hypoglycemia unawareness develops only after recurrent bouts of hypoglycemic attacks.^18^ Luckily this can be reversed by scrupulous avoidance of hypoglycemia in daily living. At least a 3-week period of meticulous avoidance of hypoglycemia should be attempted with the goal of encouraging a return to awareness of hypoglycemia.^19^

## Q8. Can diabetes cause changes in mood and behavior? How is this related to driving?

Hypoglycemia promotes mood changes, including increased irritability and anger.^19^,^20^ Long-term consequences can include a pattern of fear of hypoglycemia with a negative impact on the patient’s health-related quality of life.^6^ Patients with type 2 diabetes may experience feelings of depression and anxiety, which may affect their driving performance.^21^

## Q9. Does hypoglycemia disrupt performance of different cognitive functions or impair nonverbal intelligence? And how does this affect driving?

Acute hypoglycemia causes progressive, reversible deterioration in cognitive function that becomes detectable with glucose below 3 mmol/L.^20^,^22^,^23^ Cognitive function does not return to normal until 40 to 90 minutes after normoglycemia has been restored.^14^,^24^

## Q10. Are there any specific obligations or recommendations that a driver needs to fulfill to ensure safe driving?

Yes, and these should be discussed by his physician and other members of the diabetic team treating the patient. Our type 1 diabetes driver should measure his blood glucose before driving and not drive if it is below 5 mmol/L^16^,^25^ and should frequently recheck his blood glucose during long-journey driving.^16^,^25^ (A 2-hour interval has been suggested by some.^7^) He should discontinue driving and consume a rapid-acting carbohydrate if hypoglycemic.^16^,^25^ He should not continue driving until 30 to 45 minutes after restoration of blood glucose, as studies have shown that cognitive function may not recover until then.^16^ A study where participants did blood glucose awareness training showed a two-thirds reduction in crashes and motor vehicle violations at long-term follow-up.^25^ Type 1 diabetic drivers should be urged to wear potentially life-saving ‘diabetes alert’ identification, which will enable the rescue team to save his life and identify his blood sugar level at the moment of the accident.

## Q11. What is the doctor’s duty when treating a type 1 diabetes driver? And what precautions are advised?

Physicians have a duty to familiarize drivers with the risk of hypoglycemia and recognize its signs and symptoms.^26^ Drivers should perform self monitoring, especially when long journeys are planned. This should be correlated with meals. Drivers should be strongly advised against risk-taking behavior. If the history is suggestive of hypoglycemic unawareness, the driver should be advised to stop driving until the condition is reversed, by scrupulously avoiding further hypoglycemia for at least 2 weeks.^27^,^28^

## Q12. What are the legal responsibilities of diabetic drivers and the doctor in relation to driver and vehicle licensing agency regulations?

The diabetic driver on insulin has a duty to inform the authorities about his diabetes. If he is on diet and/or oral hypoglycemic medication and is free of complications such as hypoglycemic unawareness, there is no need to inform the authorities. Generally, insulin-treated people are not allowed to drive heavy goods vehicles or certain passenger vehicles like buses. Approvals may be sought for exceptional cases.^29^

Although the onus is on the driver to inform, the doctor may on grounds of public interest inform the authorities if he concludes that there is a risk and if the driver cannot be persuaded to notify the authorities and to stop driving. This intention must be communicated to the patient. The driver and vehicle licensing agency also recommends discussing with the next of kin, but the patient is unlikely to agree. A second opinion may be sought; but until this is resolved, the patient should also refrain from driving.^29^ The onus seems to rest largely on the individual patient, after due advice has been availed of and discussion has taken place. The legal position of the physician who fails to notify the authorities about a recalcitrant driver in the event of an accident is still untested.^29^

## Conclusion

There is insufficient evidence to prove that diabetic drivers have higher accident rates.^30^,^31^ However, some research supports the idea that hypoglycemia significantly compromises driving performance, resulting in longer response times and lower scores in cognitive tests,^10^ which may lead to traffic violations and accidents.

## References

[1] Benton D, Parker PY, Donohoe RT (1996). The supply of glucose to the brain and cognitive functioning. J Biosoc Sci.

[2] LJaE JA (2001). Beating the blood sugar blues.

[3] Comi RJ (1993). Approach to acute hypoglycemia. Endocrinol Metab Clin North Am.

[4] Blackman JD, Towle VL, Lewis GF, Spire JP, Polonsky KS (1990). Hypoglycemic thresholds for cognitive dysfunction in humans. Diabetes.

[5] McCrimmon RJ, Deary IJ, Frier BM (1997). Auditory information processing during acute insulin-induced hypoglycaemia in non-diabetic human subjects. Neuropsychologia.

[6] Lobmann R, Smid HG, Pottag G, Wagner K, Heinze HJ, Lehnert H (2000). Impairment and recovery of elementary cognitive function induced by hypoglycemia in type-1 diabetic patients and healthy controls. J Clin Endocrinol Metab.

[7] Fruehwald-Schultes B, Born J, Kern W, Peters A, Fehm HL (2000). Adaptation of cognitive function to hypoglycemia in healthy men. Diabetes Care.

[8] Hoffman RG, Speelman DJ, Hinnen DA, Conley KL, Guthrie RA, Knapp RK (1989). Changes in cortical functioning with acute hypoglycemia and hyperglycemia in type I diabetes. Diabetes Care.

[9] Cox DJ, Gonder-Frederick L, Clarke W (1993). Driving decrements in type I diabetes during moderate hypoglycemia. Diabetes.

[10] Cox DJ, Gonder-Frederick LA, Kovatchev BP, Julian DM, Clarke WL (2000). Progressive hypoglycemia’s impact on driving simulation performance: Occurrence, awareness and correction. Diabetes Care.

[11] Cox DJ, Gonder-Frederick LA, Kovatchev BP, Clarke WL (2002). The metabolic demands of driving for drivers with type 1 diabetes mellitus. Diabetes Metab Res Rev.

[12] Weinger K, Kinsley BT, Levy CJ, Bajaj M, Simonson DC, Cox DJ (1999). The perception of safe driving ability during hypoglycemia in patients with type 1 diabetes mellitus. Am J Med.

[13] Stork AD, van Haeften TW, Veneman TF (2007). The decision not to drive during hypoglycemia in patients with type 1 and type 2 diabetes according to hypoglycemia awareness. Diabetes Care.

[14] Blackman JD, Towle VL, Sturis J, Lewis GF, Spire JP, Polonsky KS (1992). Hypoglycemic thresholds for cognitive dysfunction in IDDM. Diabetes.

[15] Sommerfield AJ, Deary IJ, McAulay V, Frier BM (2003). Short-term, delayed, and working memory are impaired during hypoglycemia in individuals with type 1 diabetes. Diabetes Care.

[16] Cox DJ, Penberthy JK, Zrebiec J, Weinger K, Aikens JE, Frier B (2003). Diabetes and driving mishaps: Frequency and correlations from a multinational survey. Diabetes Care.

[17] Cryer PE (2008). The barrier of hypoglycemia in diabetes. Diabetes.

[18] Turner BC, Jenkins E, Kerr D, Sherwin RS, Cavan DA (2001). The effect of evening alcohol consumption on next-morning glucose control in type 1 diabetes. Diabetes Care.

[19] Dagogo-Jack S, Fanelli CG, Cryer PE (1999). Durable reversal of hypoglycemia unawareness in type 1 diabetes. Diabetes Care.

[20] Deary IJ, Frier Bm (2007). Symptoms of Hypoglycaemia and Effects on Mental Performance and Emotions. Hypoglycaemia in Clinical Diabetes.

[21] Abjr RR (20002). Practical Psychology for Diabetes Clinicians. American Diabetes Association.

[22] McAulay V, Deary IJ, Ferguson SC, Frier BM (2001). Acute hypoglycemia in humans causes attentional dysfunction while nonverbal intelligence is preserved. Diabetes Care.

[23] Warren RE, Allen KV, Sommerfield AJ, Deary IJ, Frier BM (2004). Acute hypoglycemia impairs nonverbal intelligence: Importance of avoiding ceiling effects in cognitive function testing. Diabetes Care.

[24] Lindgren M, Eckert B, Stenberg G, Agardh CD (1996). Restitution of neurophysiological functions, performance, and subjective symptoms after moderate insulin-induced hypoglycaemia in non-diabetic men. Diabet Med.

[25] Graveling AJ, Warren RE, Frier BM (2004). Hypoglycaemia and driving in people with insulin-treated diabetes: Adherence to recommendations for avoidance. Diabetes Med.

[26] Distiller LA, Kramer BD (1996). Driving and diabetics on insulin therapy. S Afr Med J.

[27] Fritsche A, Stumvoll M, Haring HU, Gerich JE (2000). Reversal of hypoglycemia unawareness in a long-term type 1 diabetic patient by improvement of beta-adrenergic sensitivity after prevention of hypoglycemia. J Clin Endocrinol Metab.

[28] (2009). In the driver–s seat: Managing hypoglycemia when driving [database on the Internet].

[29] DVLA (2007). Drivers Medical Group DaVLA. For medical practitioners. At a Glance: Guide to the current medical standards of fitness to drive.

[30] MacLeod KM (1999). Diabetes and driving: Towards equitable, evidence-based decision-making. Diabet Med.

[31] Songer TJ (1989). Drivers with diabetes: Innocent until proven guilty. Diabetes Care.

